# Prognostic value of baseline alkaline phosphatase of 177Lu‐PSMA radioligand therapy in metastatic castration‐resistant prostate cancer: A systematic review and meta‐analysis

**DOI:** 10.1371/journal.pone.0307826

**Published:** 2024-12-12

**Authors:** Tianhe Zhang, Wuxue Li, Haiyang Wei, Zhiheng Huang, Junkai Yang, Hanyi Zeng, Zhiyong Zhou, Xinghua Zhao

**Affiliations:** The Second Affiliated Hospital of Zhengzhou University, Zhengzhou, China; IRCCS Ospedale Policlinico San Martino, Genova, Italy, ITALY

## Abstract

**Background:**

Current studies have shown inconsistent results regarding the impact of baseline alkaline phosphatase (ALP) levels on the prognosis of metastatic castration-resistant prostate cancer (mCRPC) patients who undergo 177Lu-prostate-specific membrane antigen (PSMA) radioligand therapy (PRLT). Therefore, a comprehensive meta-analysis is needed to clarify the implications.

**Methods:**

This study was carried out in full compliance with the PRISMA protocol 2020, and a comprehensive search was conducted through PubMed, Web of Science, and Embase for published literature up to April 1st, 2024. Random-effects models were used to assess the correlation between baseline ALP and overall survival (OS) or progression-free survival (PFS) of mCRPC patients treated with 177Lu-PRLT, with a significance level set at *α* = 0.05.

**Results:**

A total of 12 articles were included in this study. The pooled effect estimates for baseline ALP and OS were 1.134 (95% *CI*: 1.035–1.245), *I*^*2*^ = 78.7%, *P* < 0.05. Regarding baseline ALP and PFS, the pooled effect estimate was found to be 2.14 (95% *CI*: 1.232–3.718), *I*^*2*^ = 93.3%, *P* < 0.05. Subgroup analysis revealed minimal heterogeneity among articles using a cut-off value ≥220U/L when examining the association between baseline ALP and OS; whereas for baseline ALP and PFS, there was also minimal heterogeneity observed among articles that adjusted for confounders.

**Conclusion:**

This meta-analysis demonstrates a significant association between elevated baseline ALP levels in mCRPC patients prior to 177Lu-PRLT treatment and inferior OS and PFS. Timely monitoring of baseline ALP levels can provide valuable insights for clinical decision-making and patient counseling.

## Introduction

Prostate cancer (PCa) is a prevalent malignancy, affecting over 1.2 million individuals annually and resulting in more than 350,000 fatalities worldwide [1]. It ranks as the third leading cause of mortality among men in the United States [2]. At the time of diagnosis, the majority of PCa cases exhibit metastasis. Despite treatment with continuous androgen deprivation therapy, the disease continues to progress, leading to metastatic castration-resistant prostate cancer (mCRPC). Patients diagnosed with mCRPC typically demonstrate depleted levels of serum testosterone (< 50 ng/dl or 1.7 nmol/L) and persistently elevated levels of prostate-specific antigen (PSA), or progression in imaging [3, 4]. Currently, conventional treatment options for mCRPC include second-generation hormone therapy, radium-223 and chemotherapy [5]. However, there are limited therapeutic alternatives available for patients who do not respond to chemotherapy like docetaxel and whose disease progression is accompanied by elevated PSA levels [6].

Prostate-specific membrane antigen (PSMA) is a type II membrane glycoprotein that exhibits high expression in both dedifferentiated and castration-resistant prostate cancer [7, 8], rendering radiolabelled PSMA imaging invaluable for detecting metastatic prostates cancer [9]. Recently, 177Lu has emerged as an optimal choice of radionuclide for the development of effective PSMA radioligand therapy (PRLT) drugs due to its favorable properties [10]. Currently, a high-affinity radioligand labeled with 177Lu-PSMA-617 has been introduced for treating mCRPC patients and it has demonstrated promising effects [11]. Similarly, another radioligand known as 177Lu-PSMA-I&T has also exhibited efficacy in treating mCRPC patients [12].

A recent meta-analysis of 177Lu-PRLT in patients with mCRPC has demonstrated a common occurrence of PSA decline following treatment, which has been found to be associated with improved prognosis [13]. Furthermore, Bräuer et al. have revealed that baseline alkaline phosphatase (ALP) levels predict prolonged overall survival (OS) and PSA-progression free survival (P-PFS) in end-stage mCRPC patients receiving this therapy [14]. Similarly, studies investigating radium-223 treatment in mCRPC patients have explored the potential role of baseline ALP and PSA as predictors for tumor response to Radium-223, suggesting their utility in guiding clinical management strategies for prostate cancer [15].

ALP is a key enzyme that plays a pivotal role in various physiological processes within the human body [16]. It exhibits widespread distribution across multiple tissues, including bone, liver, and intestine [17]. ALP serves as an indispensable component for normal bone development and growth. An early study showed that ALP is frequently utillized as an early response marker for CRPC with bone metastases, and elevated ALP levels in the initial stages have been associated with poor OS [18]. However, there is inconsistent regarding the impact of baseline ALP levels on the prognosis of mCRPC patients treated with 177Lu-PRLT in these current studies. Therefore, the objective of this meta-analysis was to synthesize existing data to validate the influence of baseline ALP levels on the prognosis of mCRPC patients undergoing 177Lu-PRLT.

## Materials and methods

### Literature search strategy

A comprehensive search was conducted in PubMed, Web of Science, and Embase for published literature up to 1 April 2024. In order to prevent any potential omissions, we utilized a broad search strategy incorporating the terms ("metastatic castration-resistant prostate cancer" OR mCRPC) and (Lutetium-177-PSMA OR ^177^Lu-PSMA) and (“overall survival” OR OS OR survival OR “progression-free survival” OR PFS). The titles and abstracts of these articles included in this study underwent independent screening by two authors, followed by downloading of pertinent papers.

### The criteria for inclusion and exclusion criteria

The criteria for inclusion were as stated below: (1) The study provides estimates of the effect and 95% confidence interval (*CI*) of baseline ALP levels on OS or PFS in mCRPC patients. (2) The mCRPC patients includes in the study treated with 177Lu-PRLT. (3) The study must be observational, including prospective or retrospective studies.

The criteria for exclusion were as stated below: (1) Repeatedly published literature. (2) Reviews, erratum, comments and articles reporting other indicators or effects. (3) Association between ALR and PFS or outcome with mCRPC patients treated with 177Lu-PRLT was not reported. (4) Inconspicuous outcome effect. (5) Non-English Studies.

### Quality assessment and data extraction

This study was conducted in full compliance with the PRISMA protocol 2020 (S1 Table) [19]. The two authors independently screened the articles according to the strict inclusion and exclusion criteria and scored them using the Newcastle-Ottawa scale(NOS). If there was disagreement, another author evaluated the articles. NOS score quality is as follows: NOS score ≥7 is high quality, 5 ~ <7 is moderate quality, <5 is low quality [20]. Then we extracted data from the final included articles, the information is as follows: the estimate effect and 95% *CI*; first author; hormone or chemotherapy; study type; published date; population; mean/median age; cut-off value and adjusted confounding factors or not. These information was then summarized into a table.

### The selection of estimate effect and 95% *CI*

When both univariate and multivariate analyses are presented in studies regarding the effect of baseline ALP levels on OS or PFS, it is common to select the multivariate analysis results, because it adjusted some potential confounding factors. Then, some studies performed a binary logistic regression analysis with survival outcomes, where the OR was approximately equal to the Hazard Ratio(HR) [21–23]. Finally, some studies classified baseline ALP according to cut-off values, but the trend ratio was inconsistent, so we normalized the estimates and 95% *CI* for studies with cut-off values, that is low concentration vs: high concentration uniformly adjusted to high concentration vs: low concentration.

### Statistical analysis

We performed a forest plot to assess the pooled effect estimate and 95% *CI* of baseline ALP on OS and PFS. The funnel plot and Begg’s test was utilized to evaluate publication bias among the included studies. The *I*^*2*^ statistic was employed to measure the heterogeneity among the studies, with *I*^*2*^ > 50% or *P* < 0.05 indicating significant statistical heterogeneity [24]. In this study, a random effects model was used considering the intra and inter study variation. Subgroup analysis was employed to investigate the potential source of heterogeneity, and sensitivity analysis was used to assess whether the research results were reliable (for each study excluded, the pooled effect and 95% *CI* of the other studies were reliable). The statistical analyses were conducted using the software STATA version 12.1 software (Stata Corp, College Station, TX, USA), with a significance level set at *α* = 0.05.

## Results

### The literature search process and the basic information of articles included in the study

**Fig 1:**
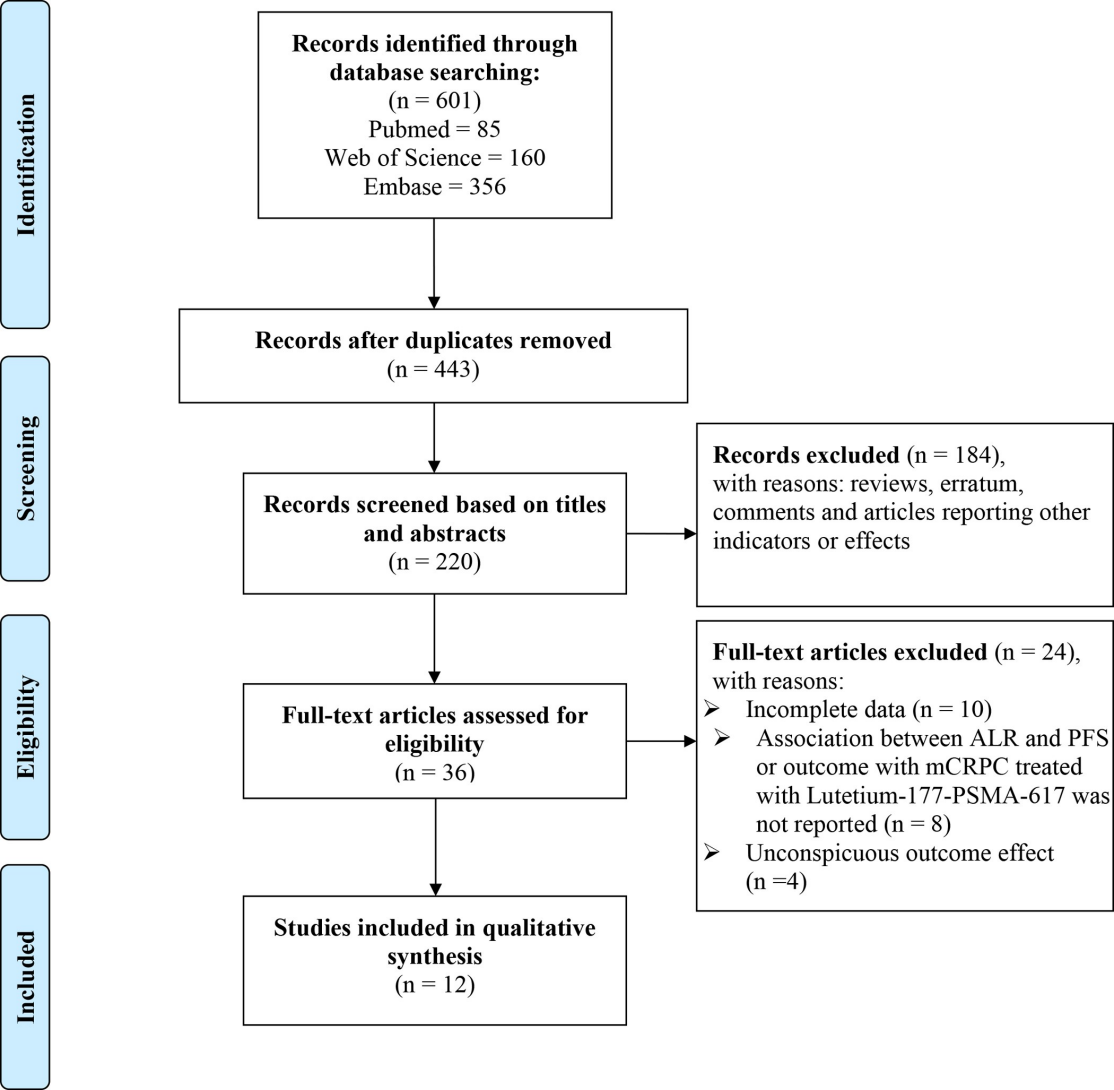
Flowchart showing the screening process for included articles.

A comprehensive search was conducted in PubMed, Web of Science, and Embase based on the specified search terms, resulting in a total of 601 records. These comprisied85 records from PubMed, 160 records from Web of science, and 356 records from Embase. Following the removal of duplicates, there were a total of 443 unique records remaining. After carefully reviewing the titles and abstracts and evaluating the remaining articles, only 12 articles were ultimately included in this study (Fig 1 and Table 1) [14, 25–35]. Among these articles, there were 10 articles on 177Lu-PSMA-617, 1 article focused on 177Lu-PSMA-I&T, and 1 article examined both therapies. Out of these articles 11 articles incorporated patients who treated with 177Lu-PRLT following hormone or chemotherapy treatment, while 2 articles did not provide this information. The majority of the articles were retrospective, with only 3 being prospective. The earliest article was published in 2017, and the primary focus of articles was on the period between 2019 and 2021. The average age of participants included in these articles exceeded 65 years old, predominantly concentrated within the range of 70 to 72 years old. 7 articles categorized baseline ALP levels using a cut-off value of 220 U/L, 1 article used a cut-off value of 333 U/L, while others reported estimates and 95% *CI* for OS or PFS for each incremental increase by either 1U/L or increments of either 50 U/L or 100 U/L. 9 studies relied on univariate analyses, whereas only 4 articles utilized multivariate analyses.

**Table 1:** Characteristics of the included articles.

Author	177Lu-PSMA Radioligand	Hormone or chemotherapy	Study type	Published date	Sample size	Mean/Median age	Cut-off Value	Effect value	Adjusted or not	Quality Scores
Khreish et al. [25]	177-Lu‑PSMA‑617	Reported	P	2022	254	70.0 (NR)	≥220 U/L	HR	adjusted	8
Barber et al. [26]	177Lu-PSMA-617, 177Lu-PSMA-I&T	Reported	R	2019	83	69.3±8.7	≥220 U/L	HR	adjusted	9
Barber et al. [26]	177Lu-PSMA-617, 177Lu-PSMA-I&T	NR	R	2019	84	70.8± 7.8	≥220 U/L	HR	adjusted	9
Bräuer et al. [14]	177-Lu‑PSMA‑617	Reported	R	2017	59	72.0 (66.0–76.0)	≥220 U/L	HR	not	9
Yadav et al. [27]	177-Lu‑PSMA‑617	Reported	P	2020	121	67.0 (60.7–72.0)	<333 U/L	HR	not	7
Rasul et al. [28]	177-Lu‑PSMA‑617	Reported	R	2021	61	71.6±6.9	1 U/L	OR	not	8
Ferdinandus et al. [29]	177-Lu‑PSMA‑617	Reported	P	2020	50	71.0 (50.0–87.0)	100 U/L	HR	not	8
Rahbar et al. [30]	177-Lu‑PSMA‑617	Reported	R	2017	104	70.0 (64.0–76.0)	≤220 U/L	HR	not	8
Khreish et al. [31]	177-Lu‑PSMA‑617	NR	R	2021	51	74.5 (46.0–89.0)	≥220 U/L	HR	not	7
Kessel et al. [32]	177-Lu‑PSMA‑617	Reported	R	2019	109	72.0 (44.7–87.5)	<220 U/L	HR	not	9
Heck et al. [33]	177Lu-PSMA-I&T	Reported	R	2018	100	72.0 (66.0–76.0)	50 U/L	HR	adjusted	9
Gaal et al. [34]	177-Lu‑PSMA‑617	Reported	R	2023	91	70.0 (65.0–76.0)	1 U/L	HR	not	7
Has et al. [35]	177-Lu‑PSMA‑617	Reported	R	2024	121	72.0 (41.0–94.0)	1 U/L	HR	not	9

Tab le footnotes: NR, not reported; P, Prospective; R, retrospective

### The pooled effect estimates and 95% *CI*

**Fig 2:**
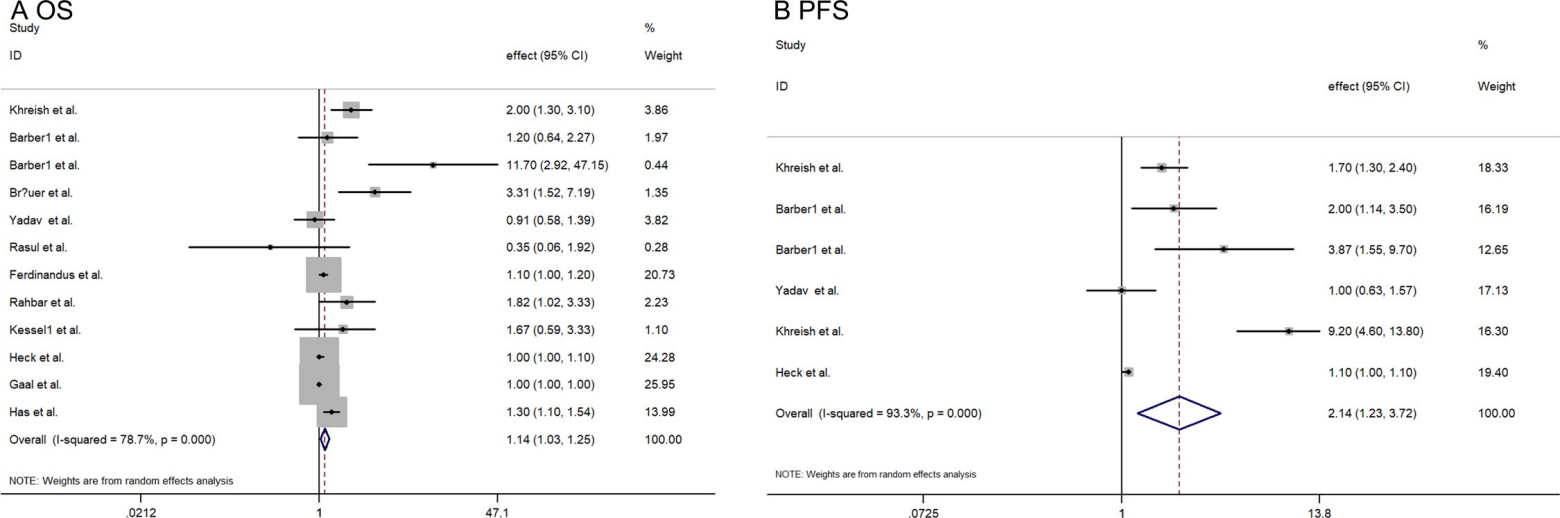
Forest plot showing the effect estimate and 95% *CI* of between baseline ALP levels on the prognosis of mCRPC patients undergoing 177Lu-PRLT: A: OS; B: PFS.

According to various clinical outcomes, we estimated the impact of baseline ALP on OS or PFS in patients with mCRPC after 177Lu-PRLT treatment. As depicted in Fig 2A and S2 Table in S1 File, a total of 12 studies reported the association between baseline ALP and OS in mCRPC patients. The pooled effect estimate was 1.134 (95% CI: 1.035–1.245), with *I*^*2*^ = 78.7% and *P* < 0.05. Additionally, 6 studies provided data on baseline ALP and PFS in mCRPC patients, yielding a pooled effect estimate of 2.14 (95% CI: 1.232–3.718), *I*^*2*^ = 93.3%, *P* <0.05 (Fig 2B and S3 Table in S1 File).

### Subgroup analysis

**Fig 3:**
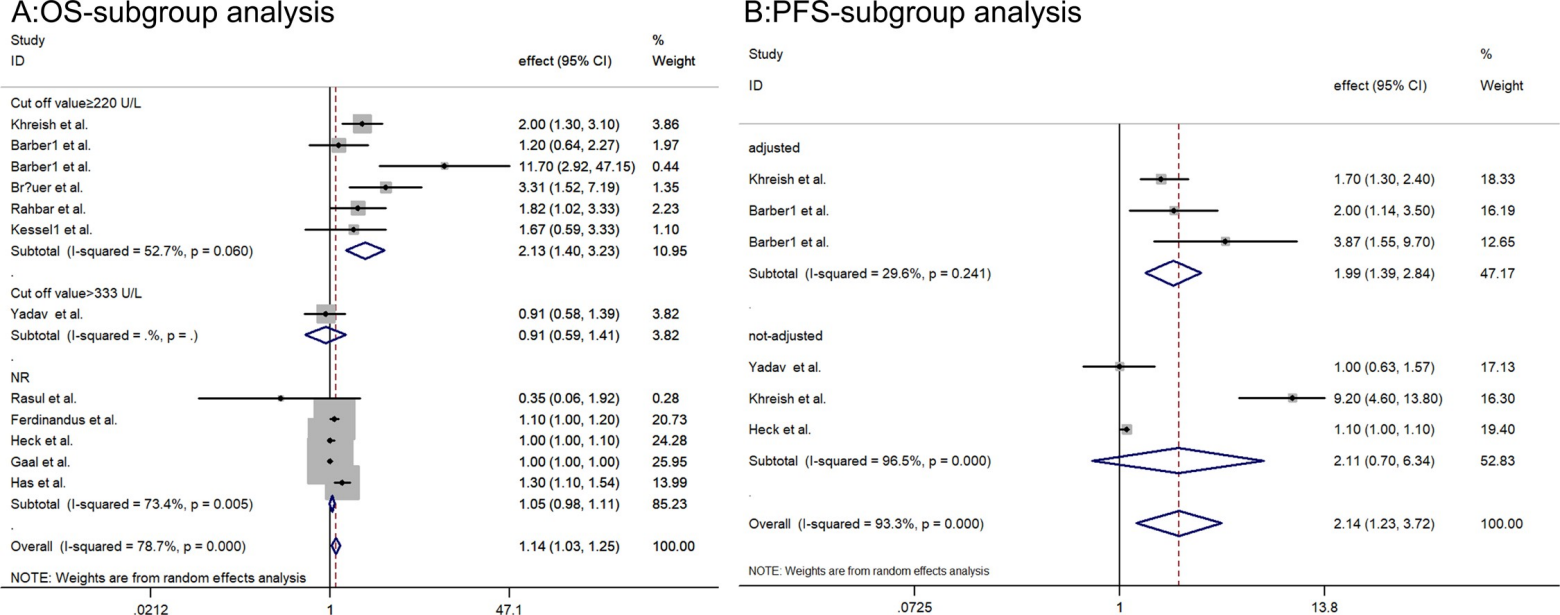
Subgroup analysis based on different variable: A: OS based on cut- off value; B: PFS based on adjusted confounding factors or not.

Given the substantial heterogeneity observed in both pooled effect estimates (*I*^*2*^ > 50%), subgroup analysis was conducted to identify potential sources of heterogeneity, primarily focusing on factors such as average age, adjusted confounding factors or not, cut-off value, effect value, population, publication date and study design. Notably, among studies assessing clinical outcome OS and grouped by a cut-off value ≥ 220U/L, minimal heterogeneity was observed (*I*^*2*^ = 52.7%, *P* = 0.06), andthe pooled effect estimate was 2.13 (1.40–3.23, Fig 3A), but no significant source of heterogeneity was identified based on other factors depicted in S1 Fig in S1 File. Similarly, for studies evaluating clinical outcome PFS, less heterogeneity existed among studies adjusted for confounders compared to those unadjusted (*I*^*2*^ = 29.6%, *P* = 0.241), and the pooled effect estimate was 1.99 (1.39–2.84, Fig 3B;), but no notable source of heterogeneity was detected based on other factors presented in S2 Fig in S1 File.

### Publication bias and sensitivity analysis

**Fig 4:**
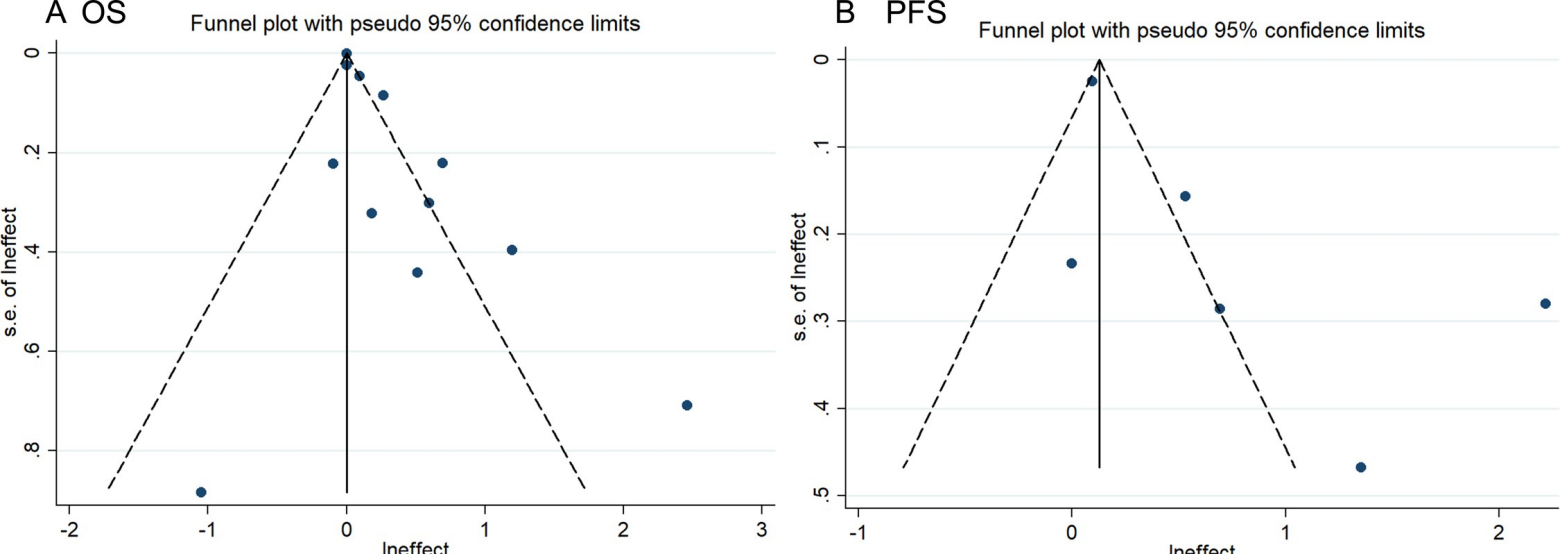
Funnel plot showing the publication bias: A: OS; B: PFS.

**Fig 5:**
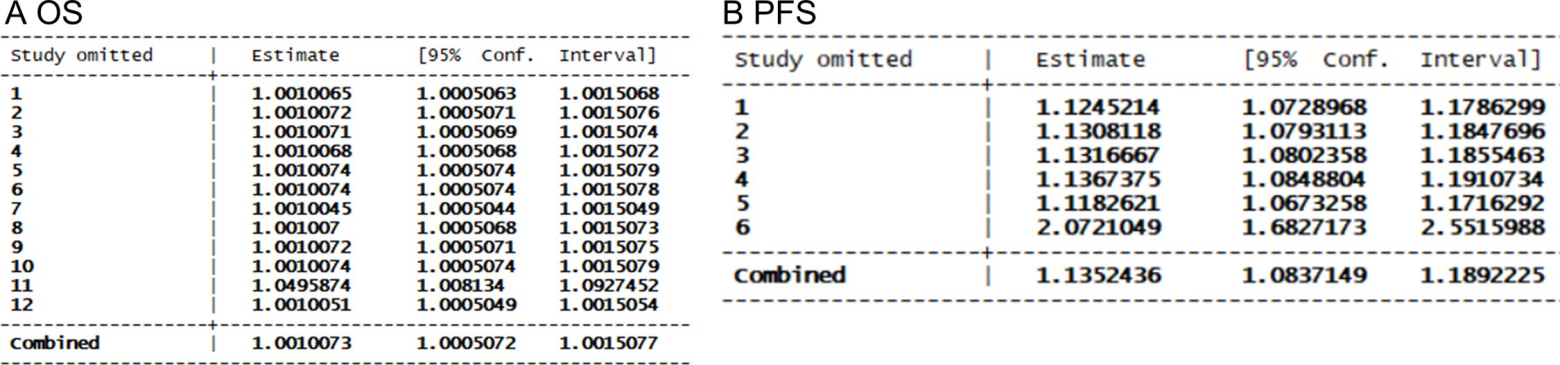
Sensitivity analysis showed the stability of the results: A: OS; B: PFS.

The funnel plot and Begg’s test were used to identify potential publication bias. Regarding the clinical outcome of OS, the funnel plot exhibited slight asymmetry, favoring a positive result in Fig 4A. However, the results of Begg’s test (t = 0.86, *P* = 0.395, S4 Table in S1 File) indicated no significant publication bias. In terms of PFS, the funnel plot displayed relative symmetry, and Begg’s test yielded non-significant results (t = 1.32, *P* = 0.188, S4 Table in S1 File), suggesting an absence of publication bias. Furthermore, to assess result stability, we conducted sensitivity analyses by excluding each study individually and examining whether it substantially affected the pooled estimates for the remaining studies included in our analysis. As shown in Fig 5, both sensitivity analyses confirmed reliable outcomes.

## Discussion

In this comprehensive systematic review and meta-analysis, we evaluated the role of baseline ALP in the prognosis of mCRPC patients after 177Lu-PRLT treatment and identified that high baseline ALP levels were a risk factor for the prognosis of OS and PFS after treatment. Recent studies have also revealed abnormal elevation of ALP levels in certain cancer types, such as breast cancer [36], lung cancer [37], gastric cancer [38], and colorectal cancer [39]. Furthermore, ALP also holds significance in the assessment of prognosis for other tumors A meta-analysis conducted by Jiang et al. demonstrated a positive correlation between elevated serum levels of ALP and bone-specific alkaline phosphatase with breast cancer metastasis and survival rates [40]. Similarly, high serum ALP levels were significantly associated with lower OS rates among osteosarcoma patients [41].

Although the prognostic value of ALP has been demonstrated in various tumor types, the underlying mechanisms remain unclear. One possible explanation is that ALP serves as a biomarker for bone metastasis, reflecting its progression when cancer begins to spread [42]. Elevated serum levels of ALP have also been observed in hormone-sensitive prostate cancer and are associated with an increased risk of overall mortality and disease progression [43]. Despite undergoing continuous androgen deprivation therapy, prostate cancer can still metastasize, with most cases progressing to mCRPC [44]. The treatment for mCRPC typically involves second-generation antiandrogens (such as abiraterone or enzalutamide), chemotherapy (including docetaxel or cabataxel), radiopharmaceuticals (like radium-223), and others [45]. A meta-analysis investigating prognostic factors in patients treated with cabataxel for mCRPC revealed that high pre-treatment ALP levels were indicative of poor prognosis [46]. Therefore, there exists a strong correlation between baseline ALP levels and prognosis in mCRPC patients.

Currently, emerging therapies are being utilized for the treatment of patients with mCRPC, and the US Food and Drug Administration has granted approval for the use of 177Lu-labeled PSMA high-affinity radioligand as a radioisotope therapy in mCRPC [33, 47]. Furthermore, treatment with 177Lu-PRLT demonstrated superior PSA response rates and fewer grade 3 or 4 adverse events compared to cabataxel [48]. The administration of 177Lu-PRLT is commonly employed in mCRPC patients who have not responded to conventional regimens like radium-223 or docetaxel, leading to significant improvements in survival outcomes with acceptable toxicity profiles [49]. Current systematic reviews and meta-analyses indicate that mCRPC patients after receiving 177Lu-PRLT treatment exhibited higher declines in prostate-specific antigen levels, lower incidence of toxicities, and prolonged OS [13, 50, 51]. However, there remained a lack of effective initial indicators to predict the prognosis of mCRPC patients treated with 177Lu-PRLT.

This study revealed a significant association between elevated baseline ALP levels and unfavorable outcomes in patients with mCRPC treated with 177Lu-PRLT, particularly in terms of OS and PFS. Despite the presence of substantial heterogeneity among the included studies, subgroup analysis demonstrated that studies employing a cut-off value ≥220 U/L exhibited minimal heterogeneity, which was more strongly correlated with poor OS. Conversely, for PFS, there was limited inter-study heterogeneity after adjusting for confounding factors. These findings are consistent with Bülbül et al.’s investigation where patients with baseline ALP≥120 U/L displayed significantly lower OS and PFS compared to those with baseline ALP<120 U/L [52]. Another study also confirmed that patients presenting baseline ALP≥220 U/L experienced shorter survival times [14]. This observation may be attributed to the potential of elevated baseline ALP levels to reflect alterations in the cellular microenvironment within injured tissues [53]. Consequently, assessing baseline ALP levels before 177Lu-PRLT treatment can offer valuable insights into treatment efficacy.

Although our findings demonstrate a significant association between elevated baseline ALP levels and unfavorable OS and PFS in mCRPC patients treated with 177Lu-PRLT, several limitations should be acknowledged: 1. Incomplete data from certain articles led to their exclusion; 2. The limited number of included articles on PFS could be one potential source of publication bias, and more studies are needed to address this issue; 3. Variations in the cut-off values for baseline ALP among the included articles resulted in substantial heterogeneity. Despite these limitations, given the relative ease of measuring serum ALP levels before treatment, large-scale prospective studies with extended follow-up periods are warranted to elucidate the potential utility of baseline ALP assessment in mCRPC patients undergoing 177Lu-PRLT treatment.

## Conclusion

This meta-analysis demonstrates a significant association between elevated baseline ALP levels in mCRPC patients prior to 177Lu-PRLT treatment and inferior OS and PFS. Notably, a baseline ALP threshold of ≥220 U/L emerges as a robust prognostic indicator for both OS and PFS. Timely monitoring of baseline ALP levels can provide valuable insights for clinical decision-making and patient counseling.

## Supporting information

S1 Table The PRISMA-2020-checklist. (DOCX)

S1 File Containing S1, S2 Figs and S2-S4 Tables. (DOCX)
