# Supplementary material for: Prognostic value of baseline alkaline phosphatase of 177Lu‐PSMA radioligand therapy in metastatic castration‐resistant prostate cancer: A systematic review and meta‐analysis
Source: PLoS One. 2024 Dec 12;19(12):e0307826. doi: 10.1371/journal.pone.0307826 (PMC11637285; doi:10.1371/journal.pone.0307826)
Supplement: S1 File — (DOCX) [file pone.0307826.s002.docx]

**Supporting information**

**Figures and tables**


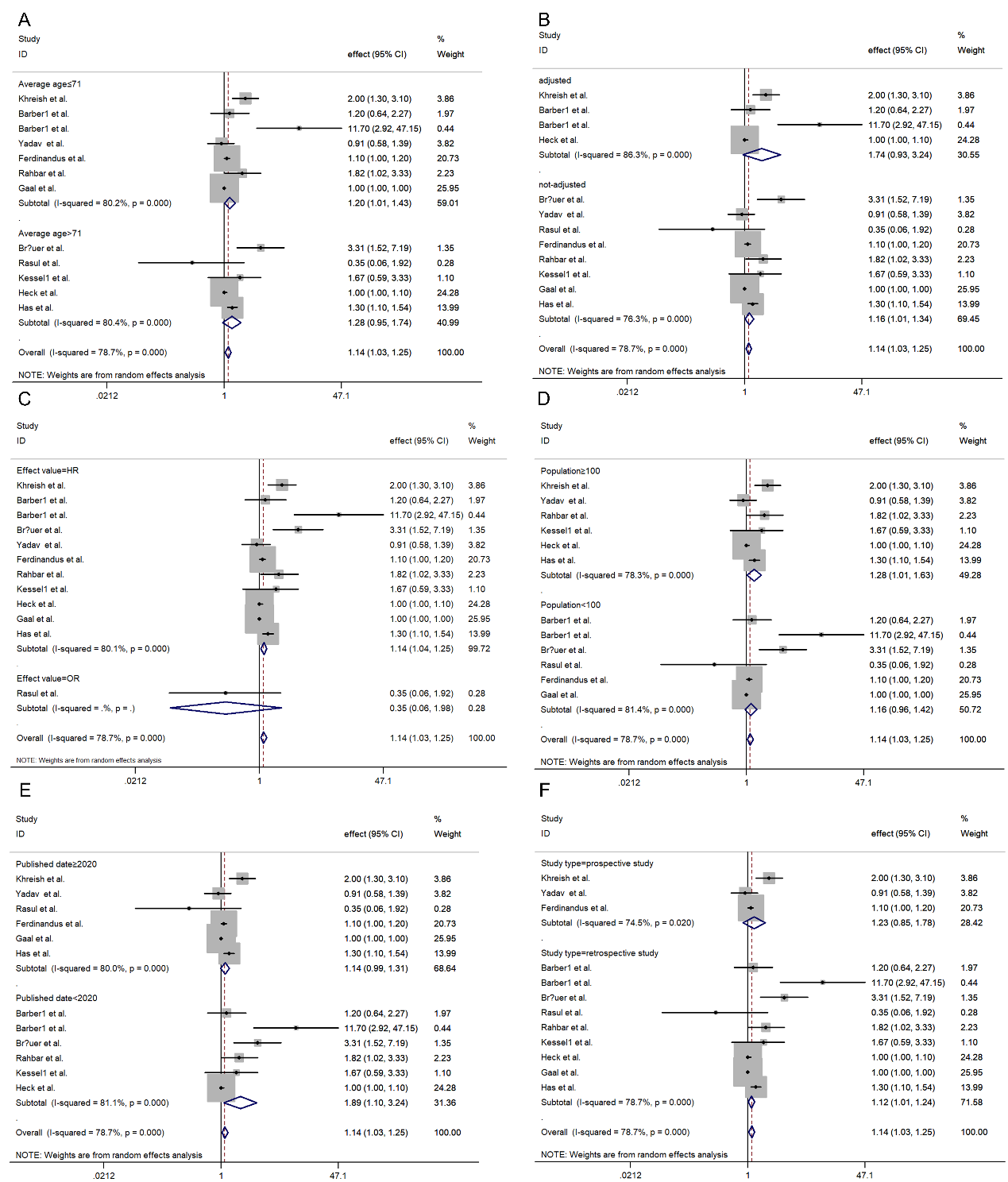


**S1 Fig** Subgroup analysis of effect estimate and 95% *CI* between ALP and OS in mCRPC patients after 177Lu-PRLT treatment (average age); B (adjusted or not); C (effect value); D (populaton); E (published date); F (study type)


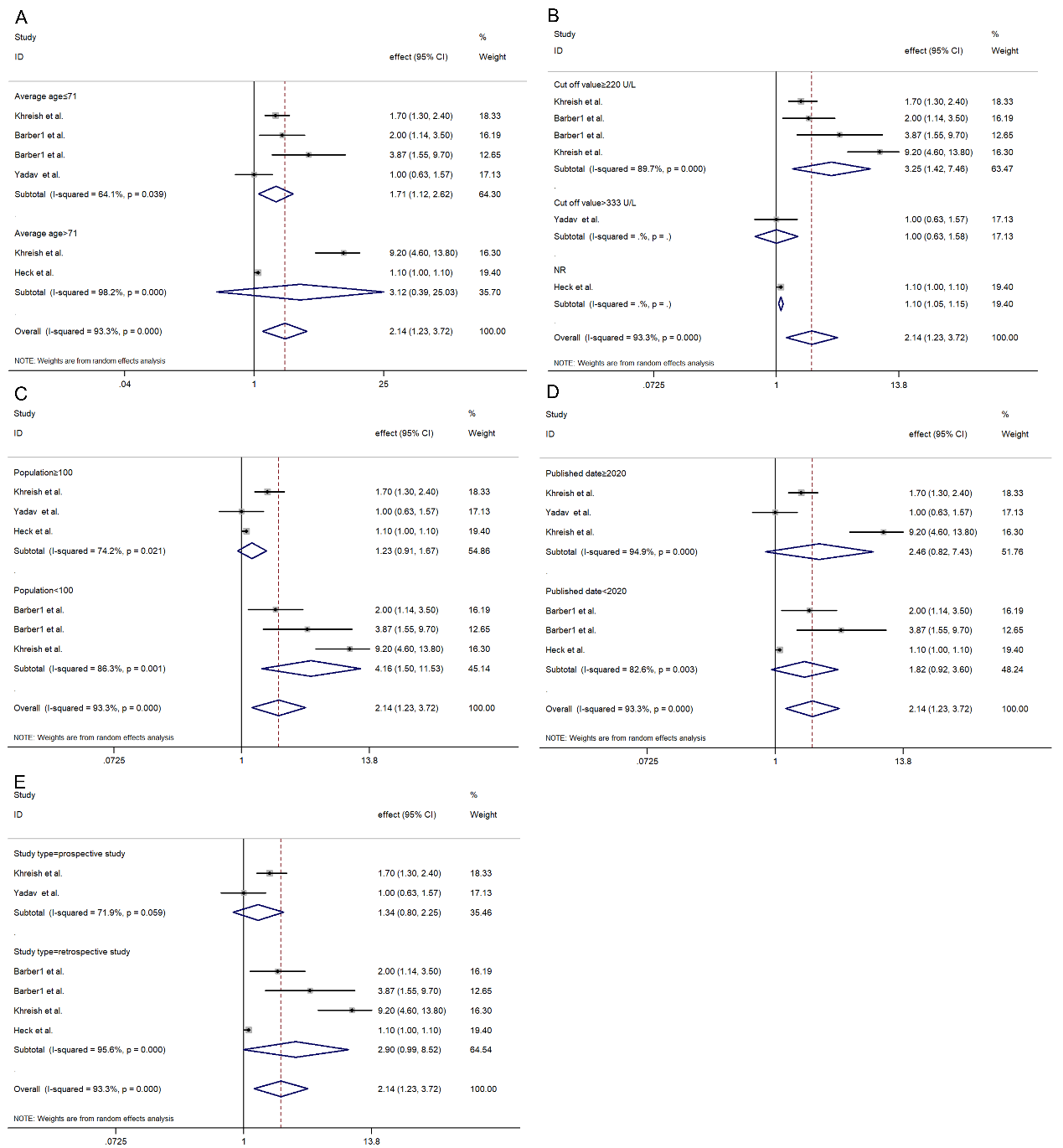


**S2 Fig** Subgroup analysis of effect estimate and 95% *CI* between ALP and PFS in mCRPC patients after 177Lu-PRLT treatment. A (average age); B (cut-off value); C (populaton); D (published date); E (study type);

.

**S2 Table** Raw data from forest plot (Figure 2A) with clinical outcomes of OS

| Author | effect | 95%CI-low | 95%CI-high |
| --- | --- | --- | --- |
| Khreish et al. | 2.0 | 1.3 | 3.1 |
| Barber1 et al. | 1.2 | 0.64 | 2.27 |
| Barber1 et al. | 11.7 | 2.92 | 47.15 |
| Bräuer et al. | 3.31 | 1.52 | 7.19 |
| Yadav et al. | 0.91 | 0.58 | 1.39 |
| Rasul et al. | 0.35 | 0.06 | 1.92 |
| Ferdinandus et al. | 1.1 | 1 | 1.2 |
| Rahbar et al. | 1.8182 | 1.0204 | 3.3333 |
| Kessel1 et al. | 1.67 | 0.59 | 3.33 |
| Heck et al. | 1 | 1 | 1.1 |
| Gaal et al. | 1.001 | 1 | 1.001 |
| Has et al. | 1.304 | 1.102 | 1.542 |

**S3 Table** Raw data from forest plot (Figure 2B) with clinical outcomes of PFS

| Author | effect | 95%CI-low | 95%CI-high |
| --- | --- | --- | --- |
| Khreish et al. | 1.7 | 1.3 | 2.4 |
| Barber1 et al. | 2 | 1.14 | 3.5 |
| Barber1 et al. | 3.87 | 1.55 | 9.70 |
| Yadav et al. | 1 | 0.6274 | 1.5699 |
| Khreish et al. | 9.2 | 4.6 | 13.8 |
| Heck et al. | 1.1 | 1 | 1.1 |

**S4** **Table** Begg’test of the overall effect and 95%CI of different outcome

| Outcome | Begg’test | |
| --- | --- | --- |
|  | t | P |
| OS | 0.86 | 0.396 |
| PFS | 1.32 | 0.188 |
